# Comparing ceramic Fischer-Koch-S and gyroid TPMS scaffolds for potential in bone tissue engineering

**DOI:** 10.3389/fbioe.2024.1410837

**Published:** 2024-08-13

**Authors:** Vail Baumer, Nelson Isaacson, Shashank Kanakamedala, Duncan McGee, Isabella Kaze, David Prawel

**Affiliations:** ^1^ Department of Mechanical Engineering, Colorado State University, Fort Collins, CO, United States; ^2^ Department of Chemical and Biomedical Engineering, West Virginia University, Morgantown, WV, United States; ^3^ School of Biomedical Engineering, Colorado State University, Fort Collins, CO, United States; ^4^ School of Materials Science and Engineering, Colorado State University, Fort Collins, CO, United States

**Keywords:** Fischer-Koch-S, gyroid, TPMS, scaffold, permeability, compressive strength, robocasting, bone tissue engineering

## Abstract

Triply Periodic Minimal Surfaces (TPMS), such as Gyroid, are widely accepted for bone tissue engineering due to their interconnected porous structures with tunable properties that enable high surface area to volume ratios, energy absorption, and relative strength. Among these topologies, the Fischer-Koch-S (FKS) has also been suggested for compact bone scaffolds, but few studies have investigated these structures beyond computer simulations. FKS scaffolds have been fabricated in metal and polymer, but to date none have been fabricated in a ceramic used in bone tissue engineering (BTE) scaffolds. This study is the first to fabricate ceramic FKS scaffolds and compare them with the more common Gyroid topology. Results showed that FKS scaffolds were 32% stronger, absorbed 49% more energy, and had only 11% lower permeability than Gyroid scaffolds when manufactured at high porosity (70%). Both FKS and Gyroid scaffolds displayed strength and permeability in the low range of trabecular long bones with high reliability (Weibull failure probability) in the normal direction. Fracture modes were further investigated to explicate the quasi-brittle failure exhibited by both scaffold topologies, exploring stress-strain relationships along with scanning electron microscopy for failure analysis. Considering the physical aspects of successful bone tissue engineering scaffolds, FKS scaffolds appear to be more promising for further study as bone regeneration scaffolds than Gyroid due to their higher compressive strength and reliability, at only a small penalty to permeability. In the context of BTE, FKS scaffolds may be better suited than Gyroids to applications where denser bone and strength is prioritized over permeability, as suggested by earlier simulation studies.

## 1 Introduction

Highly porous tissue engineered scaffolds are widely used in bone regeneration research (Roffi et al., 2017; Vidal et al., 2020) to mitigate well-publicized limitations of bone grafting methods, particularly in large bone defects, including recurring failures with non-union rates as high as 21% (Wagels et al., 2013) and complication rates of 50% due to delayed or non-union, 30% from allograft fracture, and 15% from infection (Chang and Weber, 2005). Synthetic bone tissue engineering (BTE) scaffolds attempt to accelerate the body’s natural healing process. To accomplish this, BTE scaffolds must be designed to accommodate several interrelated factors including biocompatibility, structural durability and permeability while providing a favorable environment for bone healing (Montazerian et al., 2017). Scaffolds lack the vasculature of autologous bone, emphasizing the importance of high levels of permeability, which considers porosity, tortuosity, pore size, shape, distribution and interconnectivity, key factors contributing to new bone development (Dias et al., 2012; Henkel et al., 2013), particularly in large defects. Highly interconnected, high porosity structures, within a range of 50%–90% depending on the anatomical location, are required to enable adequate mass transport of nutrients, gases and waste products during rapid bone development (Henkel et al., 2013; Wu et al., 2014). This exchange supports greater cell migration and proliferation through the three-dimensional scaffold, while playing a crucial role in angiogenesis (Klenke et al., 2008).

Calcium phosphate-based materials, such as hydroxyapatite (HAp) and tricalcium phosphate (TCP), are popular for BTE due to their biocompatibility, high levels of bioactivity (osteoconductivity, osteoinductivity and osteointegration), similarities to human bone composition, non-immunogenicity and tunable degradation rates (Hollister, 2005; Tarafder et al., 2013; Wu et al., 2014). However, these apatite scaffolds exhibit relatively low toughness due to their brittleness (Abbas et al., 2021), limiting their functional usefulness in load-bearing cases, especially as high porosity structures.

The structural integrity of BTE scaffolds can be enhanced with innovative topologies like triply periodic minimal surfaces (TPMS), which have proven to be more robust than traditional strut-based topologies (Abueidda et al., 2019; Yuan et al., 2019). They demonstrate relatively high energy absorption (Abueidda et al., 2019), complemented by high surface-to-volume ratios and an interconnected porous structure for optimal cell attachment and migration (Yoo, 2014; Alizadeh-Osgouei et al., 2021). TPMS have the capacity to be adjusted to imitate the structure of bone, thus facilitating bone growth (Alizadeh-Osgouei et al., 2021). 3D printing enables high-precision fabrication of these complex TPMS scaffolds with specific porosities, pore sizes and shapes, permeability, and tortuosity for different mechanics and applications in BTE (Alizadeh-Osgouei et al., 2021). TPMS have been 3D printed in nearly every major 3D printing process. Material extrusion with a thermal build process is most often used due to its low cost and high flexibility (Bruyas et al., 2018; Germain et al., 2018; Alizadeh-Osgouei et al., 2021). In this method a thin filament of thermoplastic is heated above its glass transition temperature and extruded from a print head that moves in three dimensions to form 3D shapes that solidify as the material cools. Robocasting is also very popular for biomimetic, ceramic scaffolds due to its very low cost and non-thermal processing method (Eqtesadi et al., 2016; Restrepo et al., 2017; Roopavath et al., 2019; Peng et al., 2021; Baumer et al., 2023). Powder bed fusion has been used to print numerous types of TPMS scaffolds (Maskery et al., 2018; Jin et al., 2022; Mulhi et al., 2023; Tilton et al., 2023), including Gyroids, in metals like titanium (Yan et al., 2015). Binder jetting has been used to print numerous types of apatite scaffolds (Butscher et al., 2011; Fielding et al., 2012; Tarafder et al., 2013; Nandi et al., 2018; Lv et al., 2019). TPMS scaffolds were also printed using vat photopolymerization (Melchels et al., 2010; Schwentenwein and Homa, 2015; Lee et al., 2021) and in hydroxyapatite- and TCP-photopolymeric slurry using digital light processing (Zeng et al., 2018; Schmidleithner et al., 2019). Recent excellent reviews discuss the advantages and disadvantages of various 3D printing processes in a wide array of materials, topologies and applications (Travitzky et al., 2014; Bose et al., 2018; Peng et al., 2018; Yuan et al., 2019).

Among the many TPMS topologies, Gyroid and Fischer-Koch S (FKS) are particularly well suited for bone regeneration scaffolds (Abou-Ali et al., 2019; Lu et al., 2020). Computational simulations have led to hypotheses that some topologies may be better suited than others in particular bone regeneration applications. For example, finite-element analysis of different TPMS topologies (Lu et al., 2020) suggest that FKS topology may be better suited to remediate a cortical diaphyseal bone defect because its high strength, low permeability, and isotropic behavior (Lu et al., 2019) better mimics dense cortical bone than other TPMS, such as Gyroids. It was similarly proposed that Gyroid scaffolds might be better suited to procedures where high anisotropy and permeability are preferable to better match the properties of cancellous bone. Gyroid TPMS have been widely studied in many forms (Restrepo et al., 2017; Bose et al., 2018; Peng et al., 2018; Abueidda et al., 2019). FKS, on the other hand, is relatively unexplored beyond computer simulations. Tools for creating G-code for printing FKS and other non-Gyroid TPMS are emerging (Baumer et al., 2023), but most are proprietary software that is bundled with expensive 3D printers. To the best of our knowledge, no one has fabricated ceramic FKS scaffolds and experimentally compared them to ceramic gyroid scaffolds. In this paper, we design and 3D print ceramic FKS scaffolds and compare key physical properties to an equivalent gyroid scaffold in the context of BTE.

## 2 Materials and methods

### 2.1 Fabrication of ceramic scaffolds

A photopolymeric resin containing 41 vol% HAp (89 nm, Macron Fine Chemicals, Avantor, Radnor, PA, United States) was created for viscous extrusion using a previously described method (Lopez, 2019). Briefly, ethylene glycol dimethacrylate (EGDMA, Scientific Polymer Products, Inc., Ontario, NY, United States) was mixed with a photoinitiator (Diphenyl (2,4,6, trimethyl benzoyl) phosphine oxide, TCI America, Portland, OR, United States), a polyanionic dispersant (Solplus D540, Lubrizol Advanced Materials Inc., Wickliffe, OH, United States), and agate milling media in a planetary ball mill (Across International, Davie, FL, United States) until a homogenous slurry resulted. Scaffolds were 3D printed on a Hyrel Engine SR 3D printer (Hyrel 3D, Norcross GA, United States) using a viscous extrusion process with simultaneous layer-wise photocuring, herein referred to as photocasting, as previously described (Baumer et al., 2023). The slurry was dispensed by a stainless-steel print head with a 0.413 mm Luer tip which was cured by an array of LED lights (405 nm wavelength) at an exposure of 0.91 mW/cm^2^. Scaffolds were removed from the build plate and sintered in a muffle furnace (Barnstead/Thermolyne 47,900, Ramsey, MN, United States) at 1,200°C for 3 h. These finished scaffolds are referred to herein as the “as-sintered” state. The homogeneity of HAp dispersion in as-sintered scaffolds was confirmed in prior work using energy-dispersive X-ray spectroscopy (EDS) (Lopez, 2019).

### 2.2 Scaffold design

3D-printable Gyroid and FKS scaffold models were developed using a previously described method (Baumer et al., 2023). The trigonometric approximations for FKS and Gyroid topologies, shown in Equations 1, 2 respectively, were used to generate a 3D non-manifold mesh using a custom open-source algorithm. Then, the surfaces were exported to Ultimaker Cura slicing software (Ultimaker B.V., Utrecht, Netherlands) to create continuous layer-wise toolpaths. One-centimeter cubes were designed with porosities of approximately 70% and pore sizes of about 1 mm for equivalent scaffolds in each topology. A single road width of 0.413 mm, equal to the nozzle diameter, was used in both designs which equates to the wall thickness. These properties are herein referred to as the “as-designed” characteristics. This process resulted in 3D-printable G-code for fabrication.

$$fks\left(x,y,z\right)=\cos\left(2x\right)\sin\left(y\right)\cos\left(z\right)+\cos\left(2y\right)\sin\left(z\right)\cos\left(x\right)+\cos\left(2z\right)\sin\left(x\right)\cos\left(y\right)=0$$ (1)

$$gyroid\left(x,y,z\right)=\sin\left(y\right)\cos\left(x\right)+\sin\left(z\right)\cos\left(y\right)+\sin\left(x\right)\cos\left(z\right)=0$$ (2)

### 2.3 Structure and surface characterization

Micro-computed tomography (micro-CT) measured the porosity, wall thickness, material spacing and surface area of as-sintered FKS and Gyroid topologies. Images were taken on a Scanco 80 (Scanco Medical AG, Bruttisellen, Switzerland) and evaluated using Scanco software with a pre-existing setting designed to scan porous, bone-like materials. The software measured the total volume (TV) of the region of interest fit to the scaffold perimeter across various slices, and then it calculated the bone volume (BV) and bone surface (BS) area of scaffold material in the domain that had a density of 903 mg HAp/cm3 or greater. The terms Trabecular thickness (Tb.Th) and trabecular spacing (Tb.Sp) within the scaffolds are used by Scanco and were calculated as defined by Scanco in the context of bone. For clarity in our study, Tb.Th refers to average wall thickness, and Tb. Sp represents the average spacing of the walls, which can be compared to average pore size. Relative porosity (φ) was determined using TV and BV as shown in Equation 3.

$$\varphi =\left(1-\frac{BV}{TV}\right)*100\%$$ (3)

Surface texture, micro-porosity, and fracture properties were evaluated on a field emission scanning electron microscope (SEM) (JOEL JSM 6500F, Peabody, MA, United States). Internal morphology was imaged from broken sections of scaffolds that were randomly selected after compression testing. Fragments were placed on the loading platform, coated with 10 nm of gold, and imaged at 10–15 kV. Fracture behavior was identified through visual examination of cracks and failure points in reference to the loading direction as indicated by the road orientation.

### 2.4 Mechanical testing

Mechanical properties of scaffolds were studied through compression tests. FKS and Gyroid cubic scaffolds were each compressed in two orientations to create four sample groups with fifteen samples per group. “Normal” referred to compression in the build direction (*Z*-axis), whereas “transverse” referred to compression orthogonal to the build direction (in the X-Y plane). Scaffold faces were smoothed with 1,200 grit sandpaper to remove protrusions which improved flush contact with the platens. Compressive stress-strain curves were obtained for each sample using a H1K-S UTM Benchtop Tester (Tinius Olsen, Horsham, PA, United States) equipped with a 1 kN load cell. Samples were placed on an aluminum crosshead and preloaded to 5 N before loading in normal and transverse directions at 0.1 mm/min crosshead speed until the applied load decreased to 25% of the peak load. Compressive testing data was imported to MATLAB (MathWorks, Inc. MATLAB R2023a) for analysis and plotting. The reliability analysis of scaffolds was conducted in Microsoft Excel to fit the ultimate mechanical strengths to a two-parameter Weibull distribution (Weibull, 1951), as described by Equation 4:

$$f\left(\sigma \right)=e^{-{\left(\frac{\sigma }{\alpha }\right)}^{\beta }}$$ (4)

where σ is the stress, and α and β are the scale and shape parameters, respectively. A Weibull probability plot was generated to determine the Weibull parameters using a least squares linear regression (Zhang et al., 2007). Once the Weibull parameters were determined for each censored or non-censored group using a Kaplan-Meier method, Monte Carlo simulations in MATLAB generated a sampling of 100,000 scaffolds to estimate the reliability of the expanded population at a given stress (Zhang et al., 2007).

### 2.5 Permeability evaluation

Permeability was assessed by correlating the fluid velocity through a cubic scaffold to a pressure gradient. If the Reynold’s number of the system is less than 1, then Stokes’ law is applicable and a Darcian flow regime (Santos et al., 2020) allows for calculation of the permeability coefficient, k, according to Equation 5.

$$k=\frac{v\mu L}{\Delta PA}$$ (5)

where v is the fluid velocity, μ is the dynamic viscosity, L is the scaffold length, ΔP is the pressure drop, and A is the cross-sectional scaffold area. Using an adaptation of the experimental setup from Santos et al. (2020), a horizontal apparatus was developed wherein a hydraulic pressure gradient was induced by flow of water through a 10 mm cubic scaffold enclosed in a 3D printed test chamber. A constant flow rate was created with a 100 mL glass syringe driven by a syringe pump (Genie Plus, Kent Scientific, Torrington, CT, United States). Flow rates of 1–5 mL/min were selected based on flows that induce wall shear stresses for optimal osteoblastic differentiation in porous scaffolds in perfusion bioreactors (Zhao et al., 2018). Components were connected with 4 mm (ID) clear Tygon^®^ tubing along with push-to-connect valves and connectors. Within the chamber, scaffolds were surrounded by an elastomeric sleeve that prevented bypass of fluid around the scaffold. A pressure transducer (Validyne Engineering, P17-16-N-1) measured the pressure differential. Based on this system design, the maximum Reynold’s number of 0.89 validated Darcian assumptions and resulted in a measurable permeability range of 1.9 × 10^−13^–6.66 × 10^−6^ m^2^, a range well suited for porous bone (Baroud et al., 2004).

The permeabilities of 5 Gyroid and 5 FKS scaffolds were calculated at flow rates of 1, 2, 3, 4, and 5 mL/min with each measurement performed in triplicate. After loading a scaffold, the circuit was purged and stabilized, then a zero-pressure reading was collected for stagnant fluid. Pressure drops in each trial were collected over a 40-s period of steady flow, and permeability was calculated using MATLAB.

### 2.6 Statistical analysis

Average values with standard deviations fit to a normal distribution are displayed in figures unless otherwise noted. Significance was determined by a two-sample equal variance, two-tailed *t*-test in MATLAB and was denoted by (*, *p* < 0.05), (**, *p* < 0.01), (***, *p* < 0.001) graphically. Mechanical comparisons between test groups featured a population of 15 samples per group resulting in 28 degrees of freedom. Darcian permeability comparisons were made between the two scaffold groups with five unique samples per group evaluated at five independently tested flowrates resulting in 48 degrees of freedom.

## 3 Results

### 3.1 Structural characterization

**FIGURE 1:**
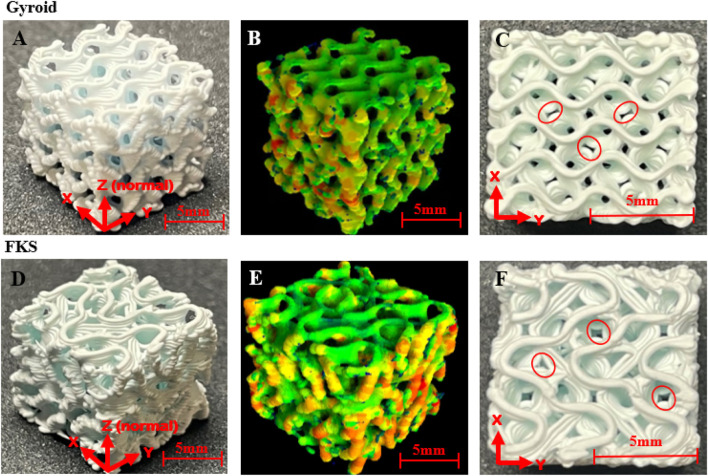
Representative images and micro-CT scans of 3D printed and sintered cubic scaffolds. The first column **(A,D)** of each row Gyroid **(A–C)**, FKS **(D–F)** compares an isometric view of each topology. The second column **(B,E)** displays 3D relative thickness “heatmaps” of each topology from micro-CT data - red is thickest, green is thinnest. The last column **(C,F)** are top views of the x-y plane where examples of layer gaps are circled.

Representative scaffold photos and micro-CT images are shown in Figure 1. Both topologies printed with comparable layer bonding, bridging, and corrugated surfaces (Figure 1A, D). 3D heatmaps from micro-CT (Figure 1B, E) highlight relative wall thickness where red areas are the thickest and green areas are the thinnest. This heatmap reveals an uneven wall thickness distribution in both structures where thicker regions can be found on the exterior vertical walls (ZY and ZX planes). FKS and Gyroid were both prone to gaps between as-printed roads (Figure 1C, F). Micro-CT analysis captured the as-sintered porosity, wall thickness, and wall spacing of FKS and Gyroid scaffolds. Average as-designed porosity (70%) of FKS scaffolds increased to 74.00 ± 0.31% as-sintered. Gyroids showed an opposite trend, decreasing to 68.49 ± 1.18%. At similar porosities, FKS scaffolds tended to have thicker walls that were spaced further apart. The average wall thickness for Gyroid scaffolds matched the extruder width of 0.413 ± 0.112 mm, where the FKS scaffolds increased to 0.424 ± 0.148 mm. Wall spacings, analogous to pore size in BTE, for FKS and Gyroid were 1.212 ± 0.295 and 1.039 ± 0.200 mm, respectively. Lastly, the mean surface area to volume ratio (i.e., specific surface area) was calculated for FKS at 5.796 ± 0.042 mm-1 and for gyroid at 5.514 ± 0.175 mm^−1^.

### 3.2 Mechanical behavior

**FIGURE 2:**
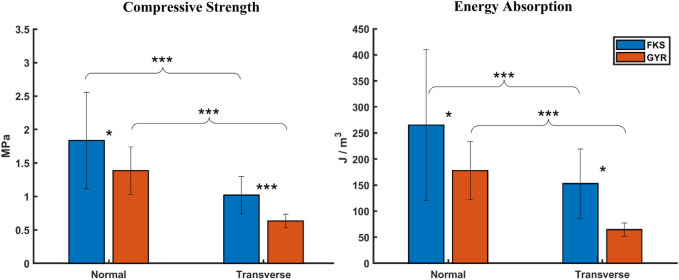
FKS scaffolds were stronger and absorbed more energy than Gyroids. Trends were similar for compressive strength and energy absorption where FKS scaffolds significantly outperformed Gyroids, and normal properties significantly exceeded transverse properties. Sample size was n = 15 for each test group. Statistical significance denoted by (*, *p* < 0.05), (**, *p* < 0.01), (***, *p* < 0.001).

Mechanical properties were calculated to quantify average scaffold performance. Four sample groups are abbreviated FKS-N, FKS-T, GYR-N, and GYR-T, where “FKS” and “GYR” refer to the topology, and “N” and “T” refer to the normal and transverse direction of compression, respectively. Compressive strength and energy absorption analysis (Figure 2) revealed that FKS scaffolds were significantly stronger and absorbed more energy than Gyroids in both tested orientations. FKS-N was 32% stronger than GYR-N with compressive strengths of 1.83±0.72 MPa and 1.39±0.35 MPa , respectively. Transverse strength was significantly weaker than normal strength for both topologies with 1.02±0.28 MPa for FKS-T and 0.63±0.10 MPa for GYR-T. Energy absorption followed the same trend where FKS-N withstood $265\pm 145\;J/m^{3}$ , a 49% increase over GYR-N, which absorbed $178\pm 56\;J/m^{3}$ . Scaffolds tested transversely were again less robust than their normal counterparts showing absorptions of $152\pm 66\;J/m^{3}$ for FKS-T and $64\pm 13\;J/m^{3}$ for GYR-T, which indicates that both of these scaffold topologies are anisotropic.

**FIGURE 3:**
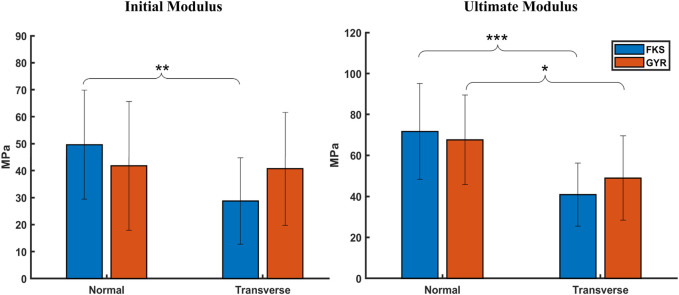
Scaffold moduli varied by testing orientation, but not by structure. No significant difference could be found between the initial or ultimate moduli between FKS and Gyroid. Scaffolds tended to be stiffer in the normal orientation than the transverse orientation except for in the initial region of Gyroids. Sample size was n = 15 for each test group. Statistical significance denoted by (*, *p* < 0.05), (**, *p* < 0.01), (***, *p* < 0.001).

Moduli were calculated using two methodologies to characterize scaffold stiffness at small strains (initial) and large strains (ultimate). No significant differences were observed between FKS and Gyroid in their initial or ultimate moduli (Figure 3). The initial strains of FKS scaffolds were notably larger in the normal direction ( $\left.49.7\pm 20.2\text{ MPa}\right)$ than the transverse direction $\left(28.7\pm 16.0\text{ MPa}\right)$ . Initial moduli of Gyroids did not significantly vary between normal and transverse testing which resulted in values of 41.8±23.9 MPa and 40.7±21.0 MPa , respectively. Ultimate moduli differed significantly between testing orientations for both topologies where FKS-N ( $\left.71.7\pm 23.3\text{ MPa}\right)$ was stiffer than FKS-T ( $\left.40.9\pm 15.4\text{ MPa}\right)$ and GYR-N ( $\left.67.6\pm 21.9\text{ MPa}\right)$ was stiffer than GYR-T ( $\left.49.0\pm 20.6\text{ MPa}\right)$ .

**FIGURE 4:**
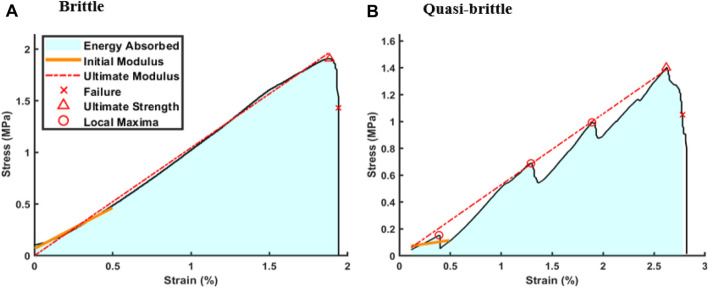
Quasi-brittle scaffold behavior. Stress-strain curves of compressed samples exhibited two distinct categories: **(A)** brittle behavior, characterized by a single linear region terminated swiftly by failure, and **(B)** quasi-brittle behavior, distinguished by multiple linear regions separated by localized failures prior to the ultimate failure.

Stress-strain curves obtained from compressive testing indicated two primary stress-strain regimes for both topologies: brittle and quasi-brittle (Figure 4). Brittle failure was represented by a stable, linear ascent to peak stress followed by abrupt failure (Figure 4A). Notably, in these instances, the initial and ultimate moduli were similar. Conversely, quasi-brittle failure was characterized by localized failures that partitioned the rise into multiple regions, frequently distinguished by distinct slopes (Figure 4B). Across both topologies and testing orientations, 45% were classified as quasi-brittle as defined by the existence of local maxima. Local maxima were recorded if a sudden decrease of 0.03 MPa or greater was observed to mark significant drops above the average level of noise. Regardless of classification, initial moduli were calculated from 0% to 0.5% strain, ultimate moduli were linearly fit from the start point to peak stress, and failure points were set at 75% of the peak load when damage was irreversible. Energy absorbed was taken as the area under the curve between start and failure. A summary of mechanical properties is included in Table 1.

**TABLE 1:** Summary of mechanical properties.

Topology	Loading direction	Ultimate strength (MPa)	Initial modulus (MPa)	Ultimate modulus (MPa)	Energy absorbed (J/m3)
FKS	Normal	1.83 ± 0.72	49.7 ± 20.2	71.7 ± 23.3	265 ± 145
Gyroid	Normal	1.39 ± 0.35	41.8 ± 23.9	67.6 ± 21.9	178 ± 56
FKS	Transverse	1.02 ± 0.28	28.7 ± 16.0	40.9 ± 15.4	152 ± 66
Gyroid	Transverse	0.63 ± 0.10	40.7 ± 21.0	49.0 ± 20.6	64 ± 13

The ultimate strength data from each scaffold group was fit to a Weibull distribution to assess failure probabilities at the low end of trabecular bone strength (0.898–29.20 MPa) (Rincón-Kohli and Zysset, 2009), as shown in Table 2. Both FKS and gyroid expressed high reliability (green) in the normal direction at loads between 1 MPa and 2 MPa, but only FKS exhibited moderate reliability (yellow) in the transverse direction. At loads exceeding 2 MPa, reliability dropped dramatically for both topologies. Interestingly, there was no measurable disparity between the strength of scaffolds showing brittle or quasi-brittle behavior. Transverse loading was notably less robust.

**TABLE 2:** Reliability analysis.

Parameters			Weibull distribution fit			Probability of exceeding	
Topology	Loading direction	Failure mode	α	β	Goodness of Fit (R^2)	1 MPa (%)	2 MPa (%)
FKS	Normal	Brittle	2.085	3.679	0.94	94	42
Gyroid	Normal	Brittle	1.576	5.577	0.95	92	2
FKS	Normal	Quasi-brittle	1.731	5.121	0.99	94	12
Gyroid	Normal	Quasi-brittle	1.992	2.706	0.89	86	37
FKS	Transverse	Brittle	1.93	1.973	0.80	76	34
Gyroid	Transverse	Brittle	0.691	8.124	0.91	0	0
FKS	Transverse	Quasi-brittle	1.167	4.018	0.89	58	0
Gyroid	Transverse	Quasi-brittle	0.761	5.257	0.99	1	0

### 3.3 Permeability

**FIGURE 5:**
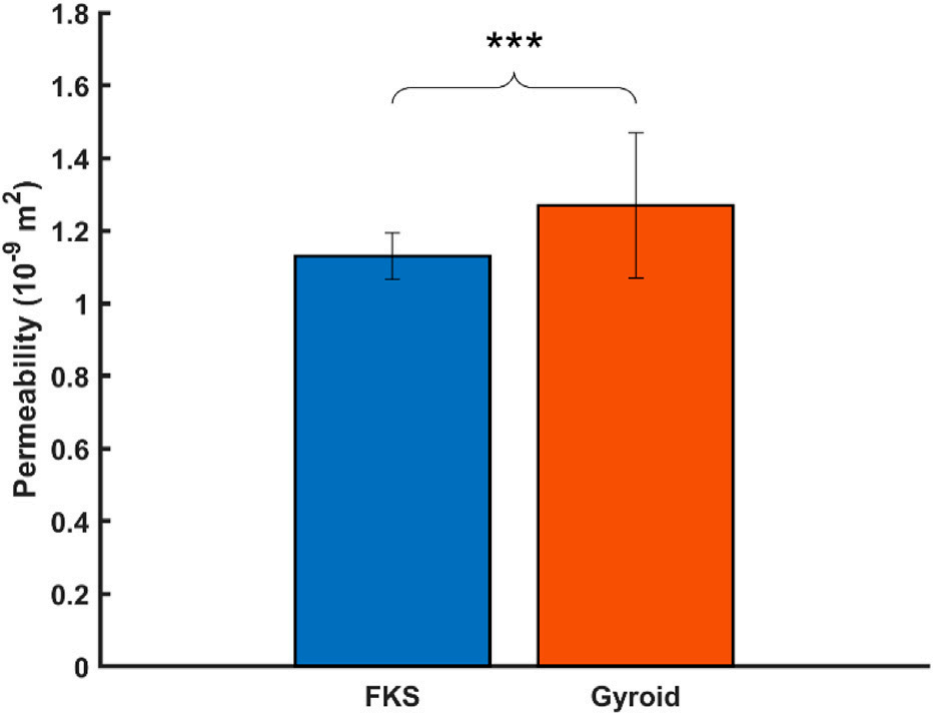
Gyroid scaffolds were more permeable than FKS scaffolds. Statistical significance denoted by (*, *p* < 0.05), (**, *p* < 0.01), (***, *p* < 0.001).

The Darcian permeability was determined by analyzing the pressure drop across each scaffold when exposed to constant flow rates typical of perfusion bioreactors (1–5 mL/min). The average permeability of FKS $\left(1.13\pm 0.06\;{*10}^{-9}\;m^{2}\right)$ was significantly lower (11%) than Gyroid $\left(1.27\pm 0.20\;{*10}^{-9}\;m^{2}\right)$ when tested in the normal direction (Figure 5).

### 3.4 Surface morphology and fracture behavior

**FIGURE 6:**
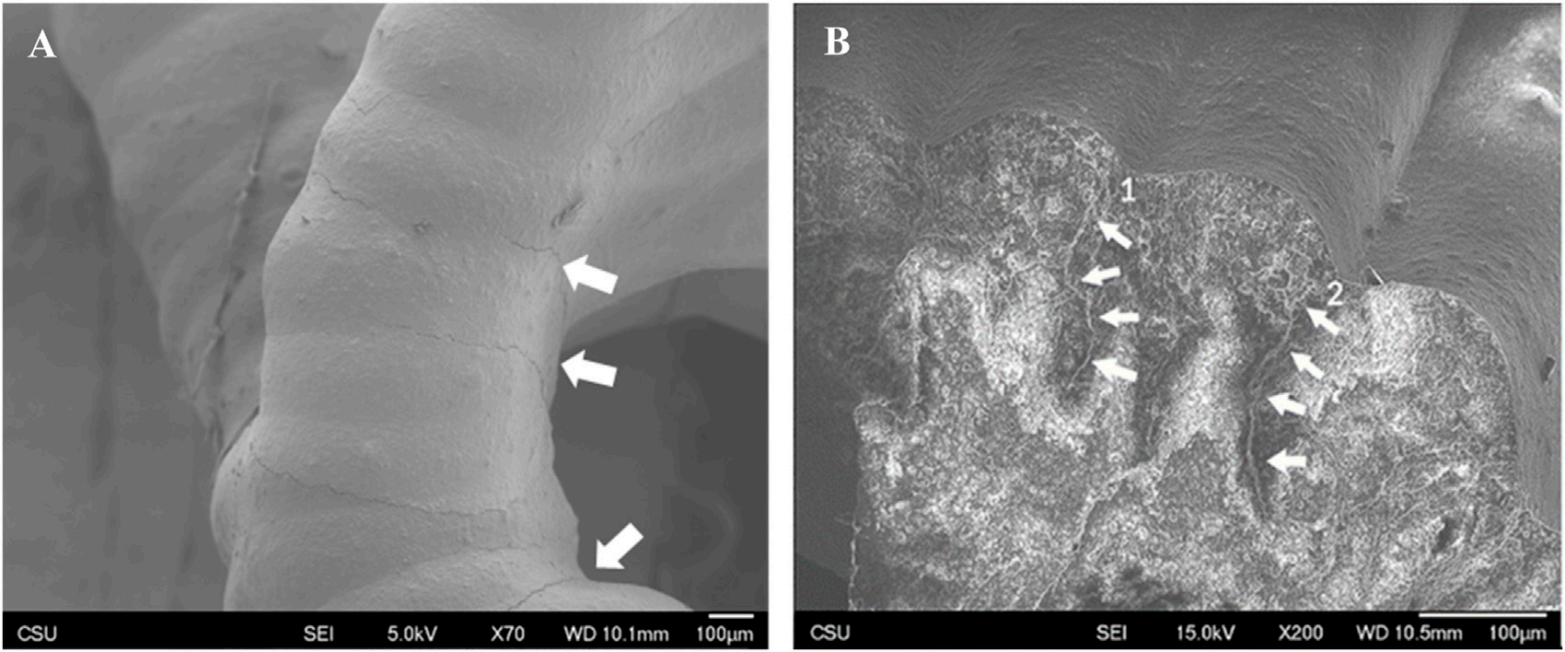
Surface texture and crack propagation. Representative images of Gyroid **(A)** and FKS **(B)** show that scaffold struts in both scaffold topologies are densely consolidated with smooth surface texture. Cracks propagated along road boundaries on the surfaces and penetrated into struts along road boundaries (B1) and (B2) when loaded in both topologies.

**FIGURE 7:**
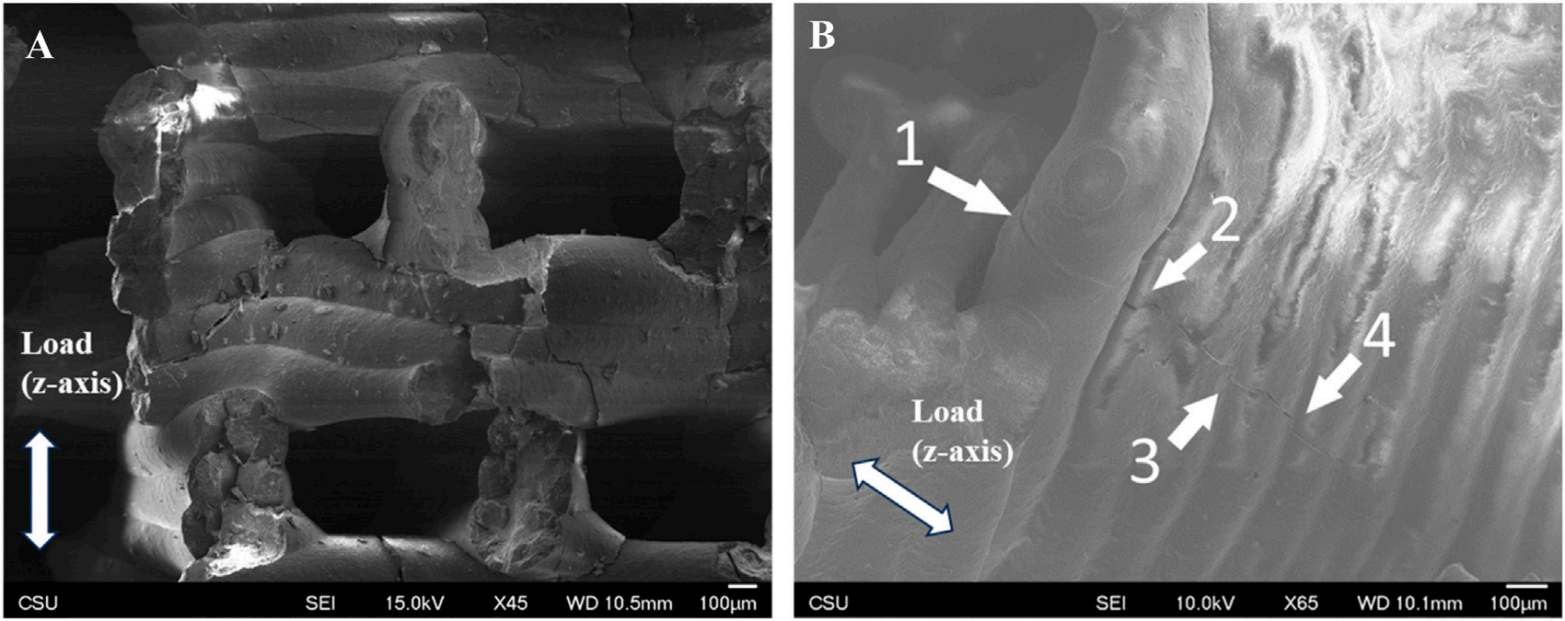
Shear failure in the direction of load. Representative SEM images of tested Gyroid **(A)** and FKS fragments **(B)** both fractured in the direction of compressive load. Image **(A)** shows shear-induced cracks at edges of open pores, and image **(B)** shows a crack that propagated through several roads, with occasional small deflections at road boundaries (B- 2, 3, 4).

Surface texture was consistent and densely consolidated in both FKS and Gyroid scaffolds. Roads were smooth, and surfaces were corrugated due to the stacking of layers. Minor surface defects were observed in both topologies, mainly initiating at road boundaries and penetrating into the scaffold (Figure 6A). Little to no evidence of elastic deformation was seen. Outer regions of road cross-sections appeared to be denser than inner regions (Figure 6B) in both topologies. In both normal and transverse loading, both scaffold types cracked internally along shear planes near the support points of suspended sections of scaffold pores (Figure 7A). Cracks propagated in the direction of load (Figure 7B), with occasional small deflections apparent at road boundaries (7B- 2, 3, 4).

## 4 Discussion

Structural characterization of each topology confirmed that robocasting produced TPMS HAp scaffolds with parameters in the nominal range for BTE in large bone defects. In terms of the optimal scaffold parameters, the critical need to balance mechanical and osteogenic properties is dependent on the material and application. In 2021, Blazquez-Carmona et al. optimized HAp scaffolds specifically for regenerating load bearing defects and provided the key insight that since these *in vivo* procedures are stabilized by metal fixation, like intramedullary nails or fixation plates, the mechanical loads required by the scaffolds significantly change (Blázquez-Carmona et al., 2021). Mechanical testing of critical defect repairs in cadaveric canine limbs have shown that the bending stiffness of the bone stabilized by an external plate did not significantly change when the scaffold was removed (Schneiderhan, 2022). This suggests that metal fixation plays a crucial role in supporting substantial loads in large bone defects, potentially enabling scaffolds to be tailored for the lower end of the bone’s strength spectrum. Considering the demonstrated benefits of higher porosity scaffolds for bone growth (Karageorgiou and Kaplan, 2005), it is preferable to pursue the highest porosity structure that fulfills the necessary mechanical failure criteria while closely mirroring the stiffness of the targeted tissue (Sturm et al., 2010). With mechanical and porosity objectives established, it is essential to recognize the impact of other design factors, such as interconnected pore size, on bone growth. While pore size studies may yield differing outcomes between *in vitro* and *in vivo* settings, there exists a widely acknowledged minimum optimum of 300 µm for scaffold design (Henkel et al., 2013; Zadpoor, 2015). Balancing pore size against factors like permeability and surface area adds complexity to the optimization process. The optimization by Blazquez-Carmona et al. recommended parameters of 59.3% porosity, 5.768 mm^−1^ specific surface area, and 360 µm pore size for their rectilinear HAp scaffolds in a large defect. Comparing optimizations proves challenging due to the diverse topologies and interrelated factors involved (Bahraminasab, 2020). Thus, considering all requirements, the TPMS scaffolds in this report were crafted with the aim of striking a balance. They were designed at the highest achievable porosity (∼70%) and pore size (∼1 mm), predicted to sustain the lower range of trabecular bone strength, as indicated by prior studies (Isaacson et al., 2022), while maintaining a specific surface area of approximately 5.7 mm^−1^. The as-sintered scaffold porosity, pore size and specific surface area values varied by topology in this report (74.00%, 1.212 mm, and 5.796 mm^−1^ for FKS and 68.49%, 1.039 mm, and 5.514 mm^−1^ for Gyroid), but they were considered good fidelity for direct comparison. Moreover, a comparison of a single high porosity design for each topology is valid in this case because trends in scaffold strength and permeability are dominated more by differences in topology than small variations (±5%) in porosity (Lu et al., 2020). Some manufacturing defects did occur, such as ooze on the exterior faces and layer gaps (Figure 1), which are believed to have caused the deviations from the as-designed 70% porosity. These gaps appear on X-Y planes consistently throughout the scaffold’s depth and are visible in sliced models in Cura prior to printing. Hence, they are attributed to interpolation of road width not the printing process. Resolution in robocasting is limited by nozzle diameter which was set to the smallest size (0.413 mm) that permitted consistent viscous extrusion of a slurry with a high solid content required to maximize mechanical strength. Since the nozzle size was fixed, and therefore the wall thickness was fixed, the FKS and Gyroid could not be reliably produced with precisely the same structural parameters because porosity, pore size, and surface area are highly interrelated. Further challenges in robocasting TPMS ceramics have been elaborated on in prior work (Baumer et al., 2023). These gaps probably reduce mechanical strength, especially in the *X*-*Y* direction, where the missing material would strengthen bonding in the internal substructure. Furthermore, these gaps increase permeability by creating additional flow paths, thereby reducing tortuosity.

The comparative properties of FKS and Gyroid scaffolds were in good agreement with current literature, and the performance of both topologies mimicked the low range of trabecular long bones. FKS scaffolds were shown to be significantly stronger and absorb more energy than Gyroid scaffolds in both normal and transverse compression, but no significant differences could be found between their moduli. In flow testing, FKS scaffolds were shown to be significantly less permeable than Gyroids scaffolds in the Darcian regime. Ravichander et al. (2022) showed that FKS scaffolds with a linear porosity gradient manufactured using metal powder bed fusion (PBF) had higher compressive strength, Young’s modulus, and energy absorption than Gyroids. Zou et al. (2022) manufactured TPMS scaffolds using titanium PBF to show that FKS had a stiffer elastic modulus, greater compressive strength, and lower permeability than Lu et al. (2020) demonstrated that FKS and Gyroid had similar compressive moduli when simulated using FEA and tested using titanium PBF across a range of porosities. In the same report, CFD calculations using both Darcy’s law and Kozeny-Carman’s relation revealed that FKS had the lowest permeability of all structures tested (including Gyroid), leading them to the conclusion that FKS may be the most favorable in scenarios where nutrient is not limiting, e.g., in bone fusion. Kapfer et al. (2011) predicted using FEA that Gyroids have a larger Young’s Modulus, but that FKS is more isotropic. Asbai-Ghoudan et al. (2021) showed that FKS had lower permeability than Gyroid across a range of pore sizes and porosities. Lu and their team also predicted FKS to be more isotropic than Gyroid using FEA, and consequently, they proposed that FKS might be better for compact bone scaffolds, while Gyroid might be better for trabecular bone scaffolds (Lu et al., 2019). Our ceramic results are in good agreement with the trends seen between FKS and Gyroid in simulations and metal-based experiments. This current study demonstrates that the compressive strength of robocast FKS and Gyroid scaffolds (1–2 MPa) were in the low range of human cancellous bone in uniaxial compression (0.898–29.20 MPa) (Rincón-Kohli and Zysset, 2009), and that their permeabilities (1.01 × 10^−9^ m^2^ to 2.18 × 10^−9^ m^2^) were similar to trabecular bone (0.4–11 × 10^−9^ m^2^) and to other scaffold literature (Grimm and Williams, 1997; Montazerian et al., 2017). Moreover, in normal compression, both topologies showed relatively high reliability (>86%) across all failure modes at the targeted 1 MPa threshold. In addition to strength and permeability, the ability of scaffolds to match the anisotropy and modulus of surrounding tissues is important for bone growth (Henkel et al., 2013), but the variability of these metrics in this report were too high to draw significant conclusions. Unlike compressive strength, the stiffness of scaffolds in this report (40–70 MPa) failed to compare to the elastic modulus range for trabecular bone (105–1,310 MPa) (Rincón-Kohli and Zysset, 2009). The prevalence of quasi-brittle behavior in nearly half of all scaffolds reduced the moduli by extending the region of strain. While this may offer advantages in terms of energy absorption and robustness, it diminished the stiffness of the constructs and introduced notable variability. These findings suggest that Gyroid and FKS HAp scaffolds may both be well suited for use in large bone defects, and that robocasting enables the structural and mechanical properties to be tuned for this specific application. FKS scaffolds show greater promise than Gyroid in mimicking natural trabecular bone strength in a high porosity ceramic due to their higher reliability in multiple loading directions. In designing BTE scaffolds, FKS scaffolds may indeed be better suited to applications of dense bone than Gyroids where strength is prioritized over permeability, as suggested by Lu et al. (2019) in earlier simulations.

In order to analyze the performance and reliability of robocast TPMS scaffolds, it was necessary to define a more robust method for determining mechanical properties to accurately characterize the mixture of brittle and quasi-brittle behavior (Figure 4). Traditional methods, such as the ASTM standard for advanced dense ceramics (ASTM C28 Committee, 2024), describe a model for “isotropic, homogeneous, and continuous behavior” which does not describe the porous HAp scaffolds tested. Unlike conventional elastic modulus analysis, stiffness calculations in this study presented a non-trivial challenge due to quasi-brittle failure. Morgan and Keaveny (2001) showed that moduli varied significantly based on the strain range chosen for bone tissue and that non-linear behavior existed even at small strains. They recommended that a second order polynomial fit be used in the region from 0%–0.2% strain for a more robust stiffness definition, but the addition of this parameter makes comparison with scaffold literature and different behavior types more difficult. To capture the diverse scaffold behavior in the current study and to compare with more classical definitions of elastic modulus, a linear fit from 0%–0.5% strain was selected for the initial modulus because it generally showed the lowest standard deviation across all sample groups. Limiting the linear fit region to an excessively small range results in high variation due to local failures, which can create a non-sensical approximation, such as seen in the quasi-brittle behavior of Figure 4B. These early local failures could have resulted from protrusions on outer scaffold surfaces due to improper sanding, but they could not be differentiated from internal local failures which are implicit in the scaffold fabrication process. As a result, no outliers were rejected, and the same rule for initial modulus was applied to all samples. It is important to note that this initial modulus does not represent the overall load-bearing capacity of the cellular scaffold due to failures and possible self-reinforcement (Isaacson et al., 2022) of internal struts, which result in multiple linear regions on the stress-strain curve before ultimate compressive strength is reached. For this reason, “ultimate” modulus was defined to represent the pre-failure stiffness of the scaffold to better characterize the functional performance of the construct in a large bone defect. Innovations in fabricating high porosity TPMS ceramics are needed to reduce variability, shrink the gap between initial and ultimate moduli, and mitigate local failures for higher performance. Despite these shortcomings, this report shows that robocast ceramics are candidates for further study in potential for aiding large bone defect repair.

The combination of brittle and quasi-brittle failures highlighted in this report raises inquiries regarding the performance of TPMS scaffolds as ceramics. Meille et al. discovered that beyond a critical porosity threshold of approximately 50%, the compressive behavior of gelcast alumina scaffolds underwent a notable transition (Meille et al., 2012). Instead of exhibiting brittle failure characterized by a linear region and abrupt collapse, the scaffolds adopted a more cellular-like fracture mode, marked by localized drops. They attributed this shift to the fact that highly porous, cellular ceramics tend to fail through individual walls rather than the propagation of cracks between isolated pores. It was additionally observed that the thickness of the wall, intricately linked to the ratio of porosity to pore size, wields considerable influence over the failure patterns, with mechanics describable through Weibull theory. Similarly, Genet et al. (2013) observed a comparable phenomenon in their rectilinear robocast HAp scaffolds. They noted that non-critical rods led to local failures, which they identified as instances of quasi-brittle behavior, analogous to cellular failure. They demonstrated a direct correlation between the Weibull coefficients and the diameter of the rod to elucidate failure behavior. Interestingly, FKS and Gyroid scaffolds in the current study exhibited this cellular/quasi-brittle behavior approximately half of the time, and the distinct failure modes did not show a clear correlation with the scaffold topology. This suggests the possible existence of a “critical” inflection point at a porosity level of 70% for this specific material and fabrication method. The Weibull theory effectively captured the failure behavior of these scaffolds across various regimes, as evidenced by high coefficients of determination. It is postulated that, even with the utilization of advanced TPMS topologies, the fracture mechanics of ceramics at high porosities remain predominantly governed by material properties and the volume of solid walls or struts.

To better understand the behavior of cellular ceramics, it is critical to examine their failure modes. SEM imagery showed (unsurprisingly) that most cracks initiated at the interfacial boundary of roads. It is hypothesized that the layer-wise photocuring process prevented homogeneity among roads due to variance in parameters like wall thickness. Corrugated walls resulting from the viscous extrusion process (Baumer et al., 2023) created thinner walls between roads and therefore stress concentrations. Not only are these road boundaries thinner, but it is predicted that the curing varied as well. Ideally, a photocast road is mostly cured during deposition, enough to bridge open pores, and is then continuously cured by subsequent exposure passes. This can result in inconsistent curing between upper and lower regions of a road and between successive roads, which was not part of this study. It is hypothesized that this resulted in interfacial defects during sintering because stresses caused by thermal gradients and variance in shrinkage dissipated through these discontinuities. Cracks tended to propagate along grains rather than through them as they follow natural crack propagation pathways. From the relatively small size of HAp grains (87 nm), there was little fracture resistance, and SEM images did not reveal significant crack deviation. Such crack propagation behavior leads to a hypothesis that initial localized failure (Figure 4B) should immediately lead to overall failure of the scaffold, but this is not always the case. The scaffolds generally failed along shear planes near the support points of suspended sections of the scaffold (Figure 7A). Due to the lattice-like nature of FKS and Gyroid structures, opportunities for the isolating the failure of non-critical rods arose where cracks extended through the entirety of a scaffold section yet the section remained in contact with the previously connected structure (Figure 7B), similar to behavior observed in prior work (Isaacson et al., 2022). As these sections of roads fail and are shifted in the direction of the load force, they can become lodged against still-intact sections of the scaffold, thereby reinforcing the strut’s integrity. Comprehending this self-reinforcing behavior is pivotal for the progress of TPMS ceramics, given their distinct failure characteristics compared to existing simulations and metal-based experiments.

It follows from these observations that all scaffolds of both topologies were weaker transversally due to the alignment of road boundaries and direction of applied loads. It is evident that anisotropy primarily resulted from layer-wise fabrication which made topological effects insignificant. The more irregular cross sections of FKS scaffolds (Baumer et al., 2023) may have contributed to their increased strength in a ceramic because cracks required more deflection to become catastrophic, and opportunities for self-reinforcement were greater. Previous work in our lab has shown similar self-reinforcing behavior in Gyroid HAp scaffolds at various porosities (Isaacson et al., 2022). One key observation in both studies is a slightly negative correlation between compressive strength and the propensity for local failures. Generally speaking, stronger scaffolds failed catastrophically whereas comparatively weaker scaffolds showed quasi-brittle behavior as described by the small differences in reliability in Table 2. This makes intuitive sense; Quasi-brittle behavior necessitates the initial failure of weaker walls or layers, a phenomenon likely attributable to manufacturing defects that serve as sites for crack initiation. Preventing the formation of the microcracks that cause early failure has been discussed for quite some time (Lankford et al., 1998). While methods developed to model this failure in comparatively geometrically simple scaffolds have previously been investigated (Genet et al., 2013; Gross et al., 2019), modeling of more complex TPMS ceramic structures is limited. Due to the comparatively young age of additive manufacturing, empirical evaluation centered on road size of ceramic scaffolds has only relatively recently begun (Sabree et al., 2015; Thiraux et al., 2022).

## 5 Conclusion

In this study, FKS and Gyroid scaffolds were photocast in HAp to compare experimental properties for their use in BTE. Bone regeneration scaffolds face a pivotal design challenge in striking a balance between strength and permeability, with potential enhancements attainable through the utilization of TPMS structures. We presented the initial elastic modulus of these structures to enable more classical comparisons with other structures and materials and in addition we argue that current methods do not adequately characterize the unique quasi-brittle behavior of TPMS ceramics. We therefore propose that ultimate modulus may be a better predictor of performance in large bone defects. Results revealed that both topologies could achieve the lower ends of the strength and permeability ranges akin to trabecular long bone, with high reliability. However, FKS exhibited notable strength advantages with only a minor compromise in permeability, compared to Gyroid scaffolds. In the context of designing BTE scaffolds, FKS structures appear to be better suited than Gyroids for further study in applications prioritizing denser bone and strength over permeability, echoing insights from earlier simulations (Lu et al., 2019). The TPMS ceramics examined in this report displayed quasi-brittle failure modes effectively described by Weibull distributions. Notably, this cellular failure mode remained consistent across different topologies and has been observed in prior studies utilizing rectilinear and gas-forming methods (Meille et al., 2012; Genet et al., 2013). This suggests that material properties and strut thickness predominantly govern ceramic failure at high porosities. The occurrence of localized failures poses a significant obstacle in accurately mimicking the elastic modulus of bone using cellular ceramics. As bone tissue engineering advances for high porosity ceramics, FKS emerges once more with promising results among the TPMS options, reinforcing its potential for applications demanding both strength and structural integrity.

## Data Availability

The raw data supporting the conclusions of this article will be made available by the authors, without undue reservation.
